# Supplementary material for: Notch-mediated inhibition of neurogenesis is required for zebrafish spinal cord morphogenesis
Source: Sci Rep. 2019 Jul 10;9:9958. doi: 10.1038/s41598-019-46067-1 (PMC6620349; doi:10.1038/s41598-019-46067-1)
Supplement: Supplementary file 1 — Supplementary Information [file 41598_2019_46067_MOESM1_ESM.pdf]

Supplementary Information

Notch-mediated inhibition of neurogenesis is required for zebrafish spinal cord morphogenesis

Priyanka Sharma, Vishnu Muraleedharan Saraswathy, Li Xiang and Maximilian Fürthauer

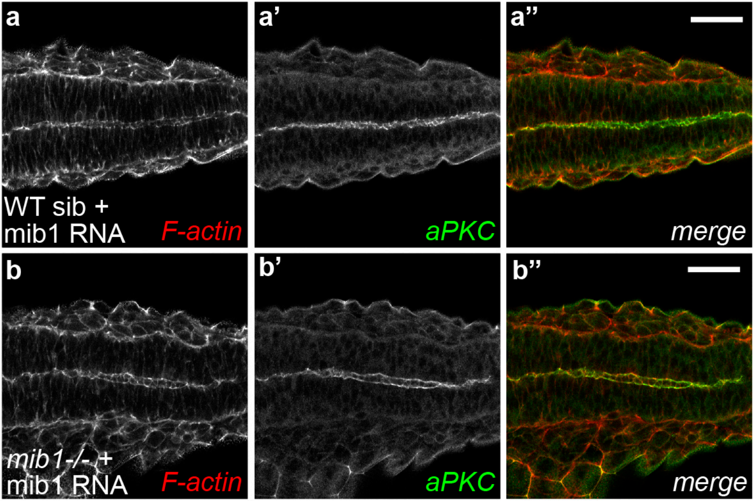

Mindbomb1 is important for zebrafish spinal cord morphogenesis
(**a,b**) Injection of RNA encoding wild-type Mib1 protein restores neuroepithelial morphology (visualized using F-actin staining) and polarized accumulation of the apical marker aPKC in *mib1* mutants (5/7 embryos full rescue, 2/7 partial rescue). Dorsal views of the anterior spinal cord at the 18 somites stage, anterior left. Scalebars: 50 µm.

Supplementary Figure S1

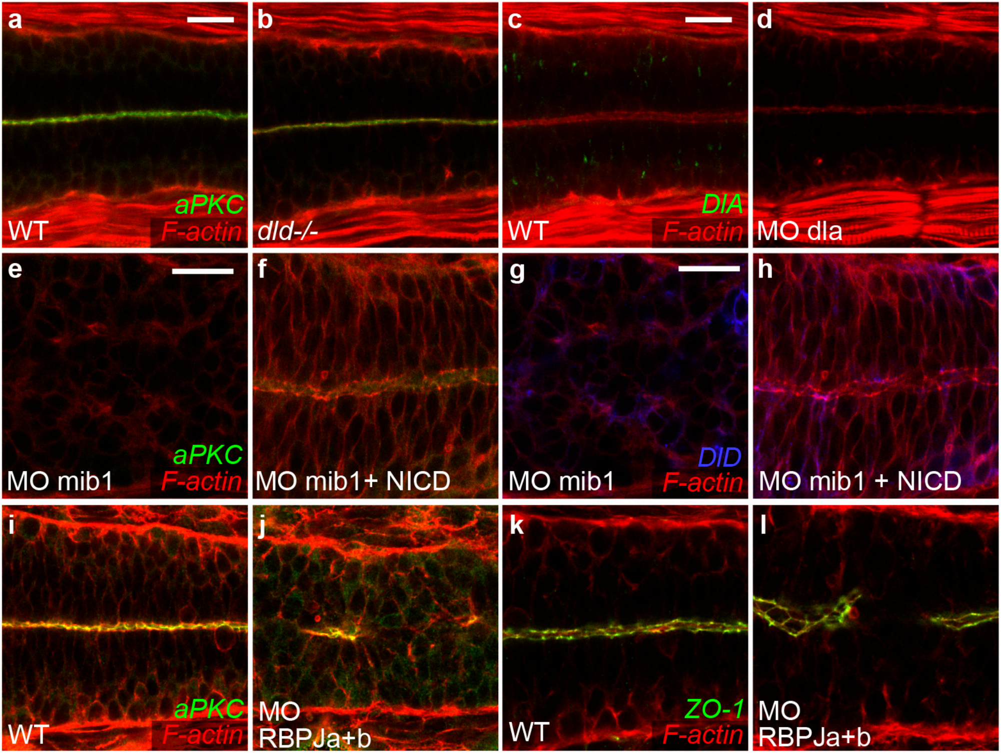

Notch pathway components are required for the apico-basal polarity of the zebrafish spinal cord (**a,b**) Apical aPKC staining and cortical F-Actin indicate normal apico-basal polarity and neuroepithelial morphology in *dld*^{AR33} mutants (n=8/8). (**c,d**) dlA morpholino injection does not alter neuroepithelial morphology but abolishes DlA immuno-reactivity (n=4/4). (**e-h**) RNA injection of a constitutively activated form of Notch (NICD) restores neuroepithelial morphology and apical aPKC localisation (**f**) but not DeltaD endocytosis (**h**) in 14/17 embryos. (**i-l**) Embryos injected with morpholinos against RBPJa & b display a partial loss of apico-basal polarity as visualized by a partial disruption of apical aPKC (**j**, n=23/29) and ZO-1 (**l**, n=16/16). (**a-d**) 26 somites stage, (**e-l**) 30 somites stage. Phalloidin staining of F-actin is used to highlight cell outlines. Scalebars: 20 µm.

Supplementary Figure S2

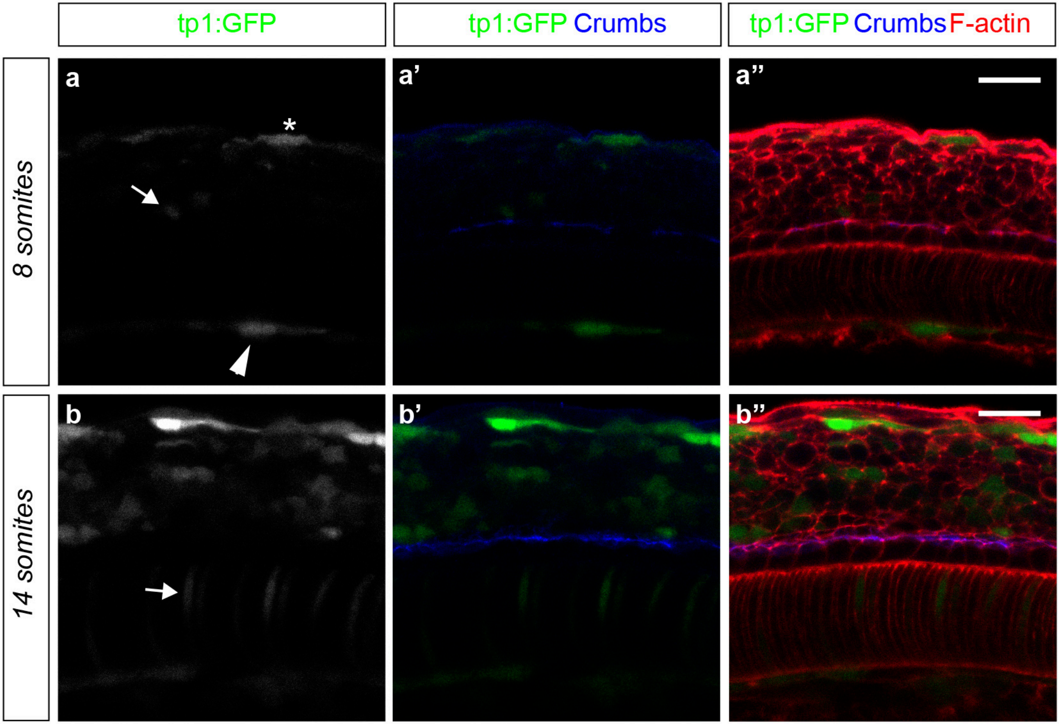

Early floor plate morphogenesis occurs in the absence of detectable Notch reporter activity
(**a,b**) Lateral views of floor plate cells at the level of the anterior spinal cord, anterior to the left, dorsal up. (**a**) At the 8 somites stage, the activity of the transgenic Notch reporter line *tp1:bglob-GFP* (tp1:GFP) is detected in isolated neural tube cells (arrow), hypocord cells (arrowhead) and epidermal cells (star) (n=13). (**b**) At 14 somites, numerous GFP-positive cells are found in the neural tube and additional reporter expression is detected in notocord cells (arrow) (n=13). Floor plate cells, which can be identified through their cuboidal morphology (F-Actin in **a'',b''**) do not display Notch reporter activity. 8 and 14 somites stage embryos were imaged using the same confocal settings. For display purposes, contrast enhancement was then used to improve the visibility of the weak 8 somites stage *tp1:GFP* and Crumbs signals in **a-a''**. Scalebars: 20 µm.

Supplementary Figure S3

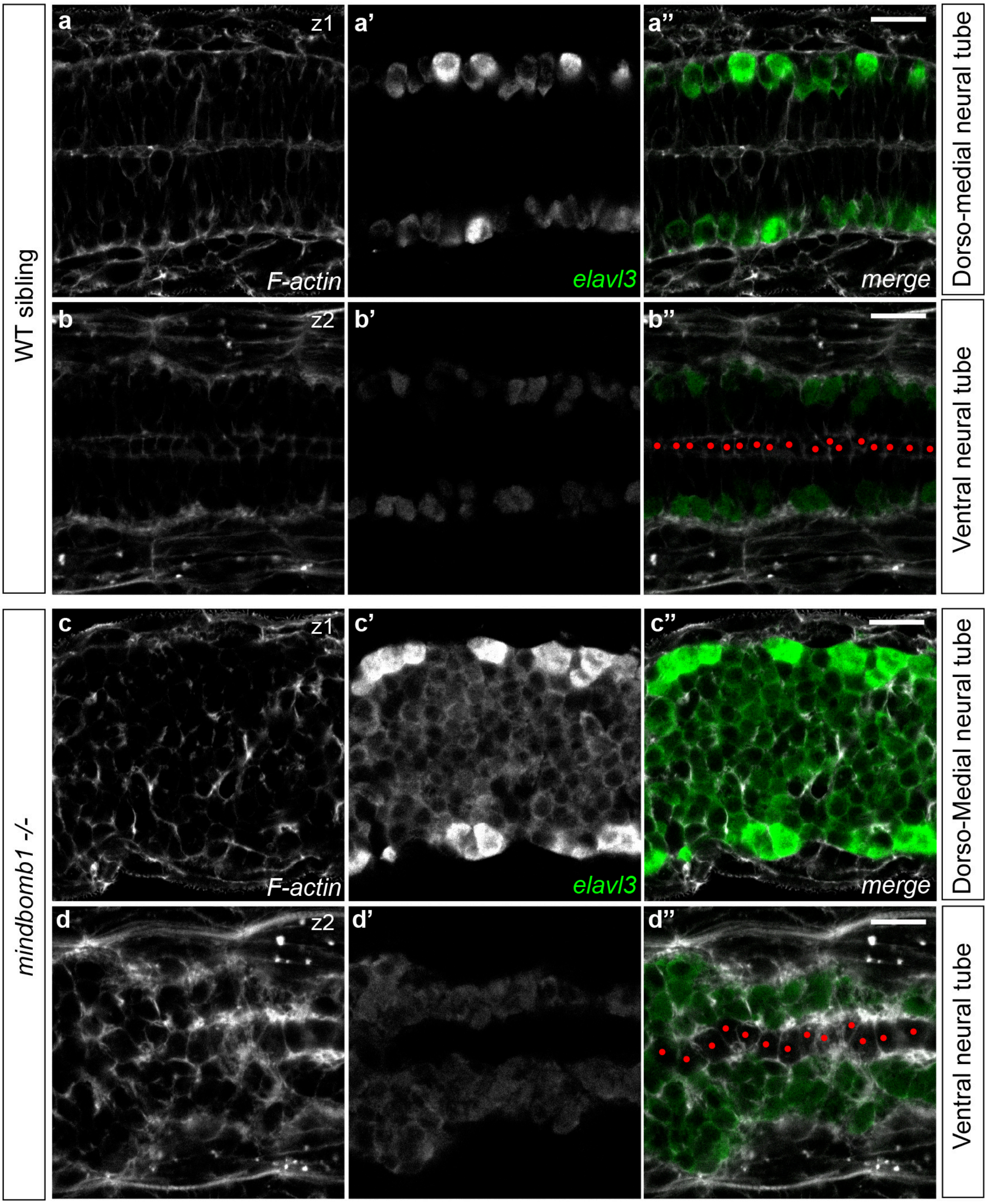

Excessive neuronal differentiation causes neural tube morphogenesis defects in *mindbomb1* mutants

(**a-d**) In 22 somites stage WT siblings, the neuronal differentiation marker *elavl3*a is expressed in some cells of the dorso-medial (**a',a''**) or ventral (**b,b''**) spinal cord. In *mib1* mutants, all dorso-medial neural tube cells differentiate as neurons (**c',c''**), causing a loss apico-basally polarized precursor cells and a disruption of neuroepithelial morphology (n=4/4). In the medial spinal cord of *mib1* mutants the elavl3 signal occupies 76.5±10.2% of the neural tube surface compared to 14.6±2.5%, in WT siblings (p=7.61E-04). In the ventral neural tube, floor plate cells can be identified by their cuboidal morphology (red dots in **b'',d''**). In *mib1* mutants, floor plate cells are the only ones not undergoing neuronal differentiation (**d',d''**, n=4/4). In this region, elavl3 occupies 60.5±0.69% of the neural tube compared to 14.2±2.5%, in WT siblings (p=1.52E-05). **a,b** and **c,d** are views of the same embryo at different z levels. All images are dorsal views of the anterior spinal cord, anterior left. Scalebars: 20 µm.

Supplementary Figure S4

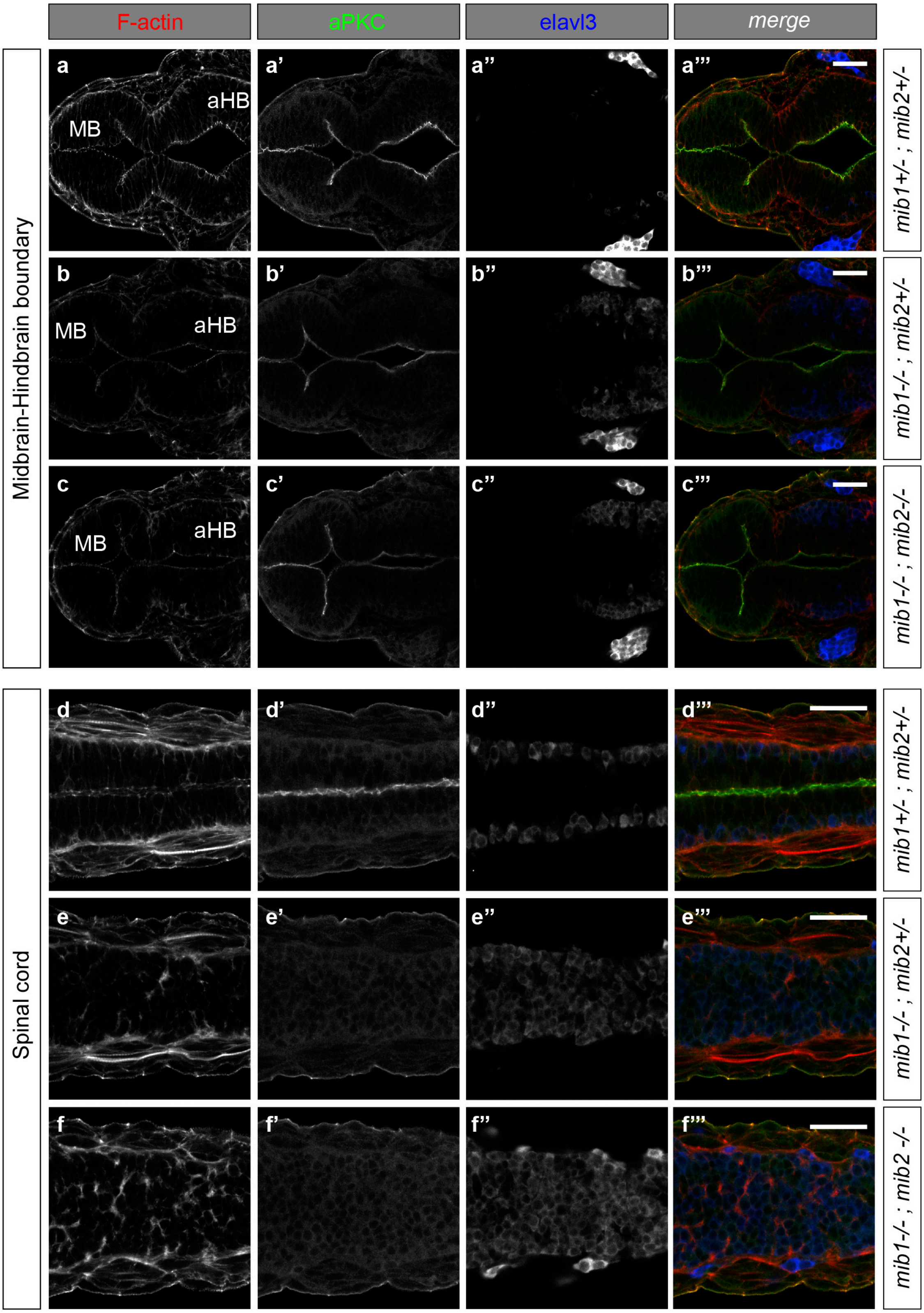

***mindbomb1* ; *mindbomb2* double mutants retain neuroepithelial polarity in the midbrain-hindbrain boundary region**
(**a-f**) Dorsal views of the midbrain-hindbrain boundary region (**a-c**) and anterior spinal cord (**d-f**) at the 30 somites stage, anterior left. (**a,d**) Neuronal differentiation (visualized using elavl3) and neuroepithelial polarity (highlighted by apical aPKC acumulation) occur normally in $mib1^{tfi91/+};mib2^{chi3/+}$ transheterozygotes (n=3, compare to WT siblings in **Fig. 6i**). (**b,c,e,f**) $mib1^{tfi91/tfi91};mib2^{chi3/+}$ (n=10) and $mib1^{tfi91/tfi91};mib2^{chi3/chi3}$ double mutant (n=6) animals present a similar increase in neurogenesis the level of the anterior hindbrain (aHB, **b”,c”**) and the anterior spinal cord (**e”,f”**). See Supplementary Table S1 for a quantification of spinal cord neurogenesis. Neuroepithelial polarity is disrupted in the spinal cord (**e’,f’**) but maintained in the midbrain (MB, **b’,c’**). (**a,d**), (**b,e**), (**c,f**) represent different regions of the same embryos. Scalebars: 40 µm.

Supplementary Figure S5

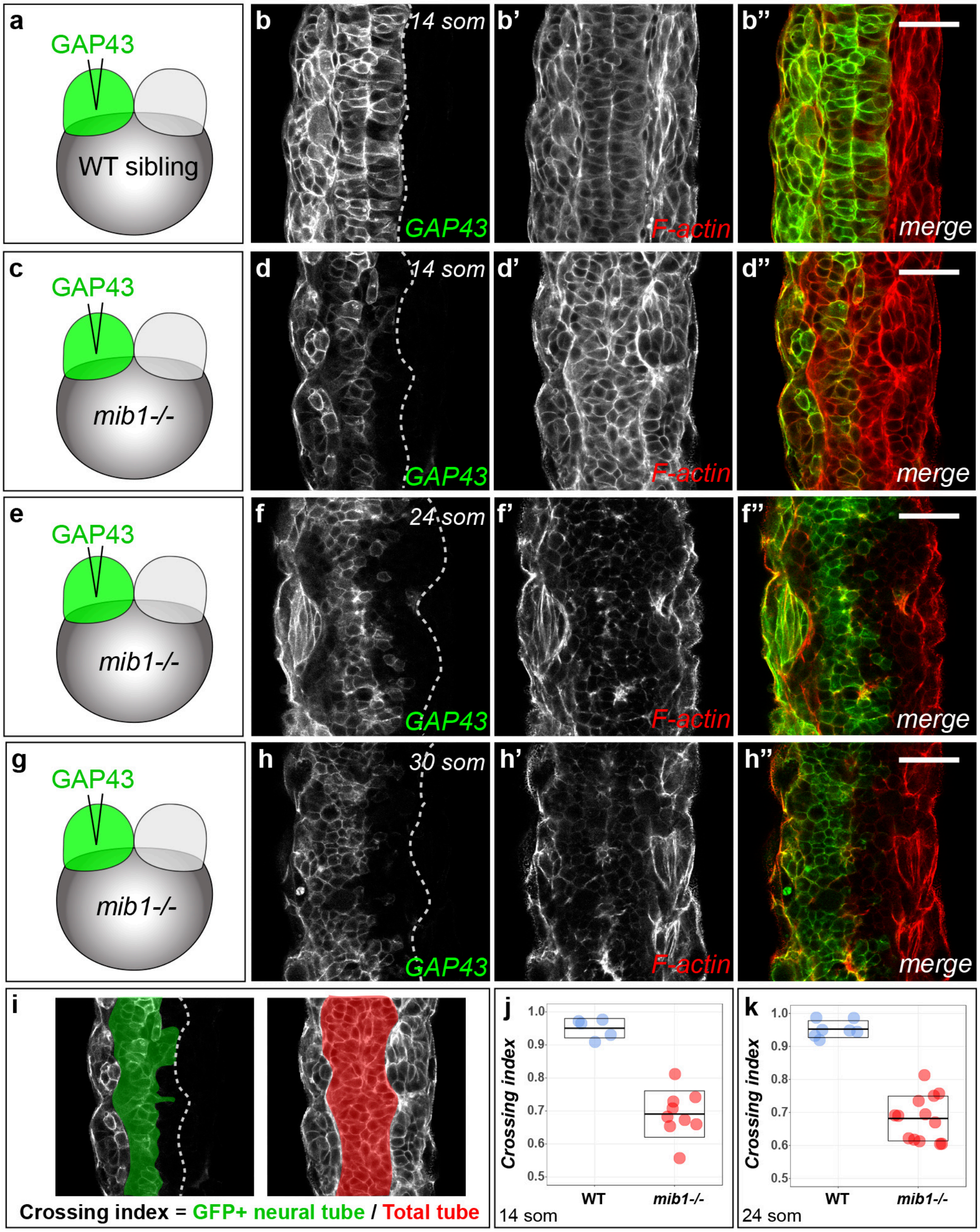

Mindbomb1 **loss of function impairs the midline-crossing behavior of neural tube cells**

(**a-j**) One half of the neural tube was labelled by injecting RNA encoding a green membrane label (GAP43-GFP) into one blastomere of 2-cell stage embryos (see Methods). (**a,b**) In 14 somites stage WT sibling embryos, cells originating from one side of the neural tube have crossed the neural tube midline to integrate the contra-lateral organ half. (**c,d**) In *mib1* mutants cells fail to cross to the contra-lateral side. (**e-h**) This inhibition of midline-crossing persists if *mib1* mutants are analysed a 24 somites (**e,f**) or 30 somites (**g,h**, see **Fig. 6l** for quantification). (**i**) For the quantification of neural tube cell midline crossing, the total area of the neural tube (red) and the area of the neural tube populated by GFP-positive cells (green) were outlined (see Methods for details). The neural tube cell midline crossing index represents the fraction of the neural tube that is occupied by GFP-positive cells. (**j**) Neural tube cell midline crossing is reduced in 14 somites stage *mib1* mutants (0.69±0.07, n=9) compared to WT siblings (0.95±0.03, n=5) (p=7.0E-07). (**k**) Neural tube cell midline crossing is reduced in 24 somites stage *mib1* mutants (0.68±0.07, n=13) compared to WT siblings (0.95±0.03, n=7) (p=4.2E-10). **(b,d,f,h,i)** dorsal views of the anterior spinal cord, anterior up. Scalebars: 40 µm.

Supplementary Figure S6

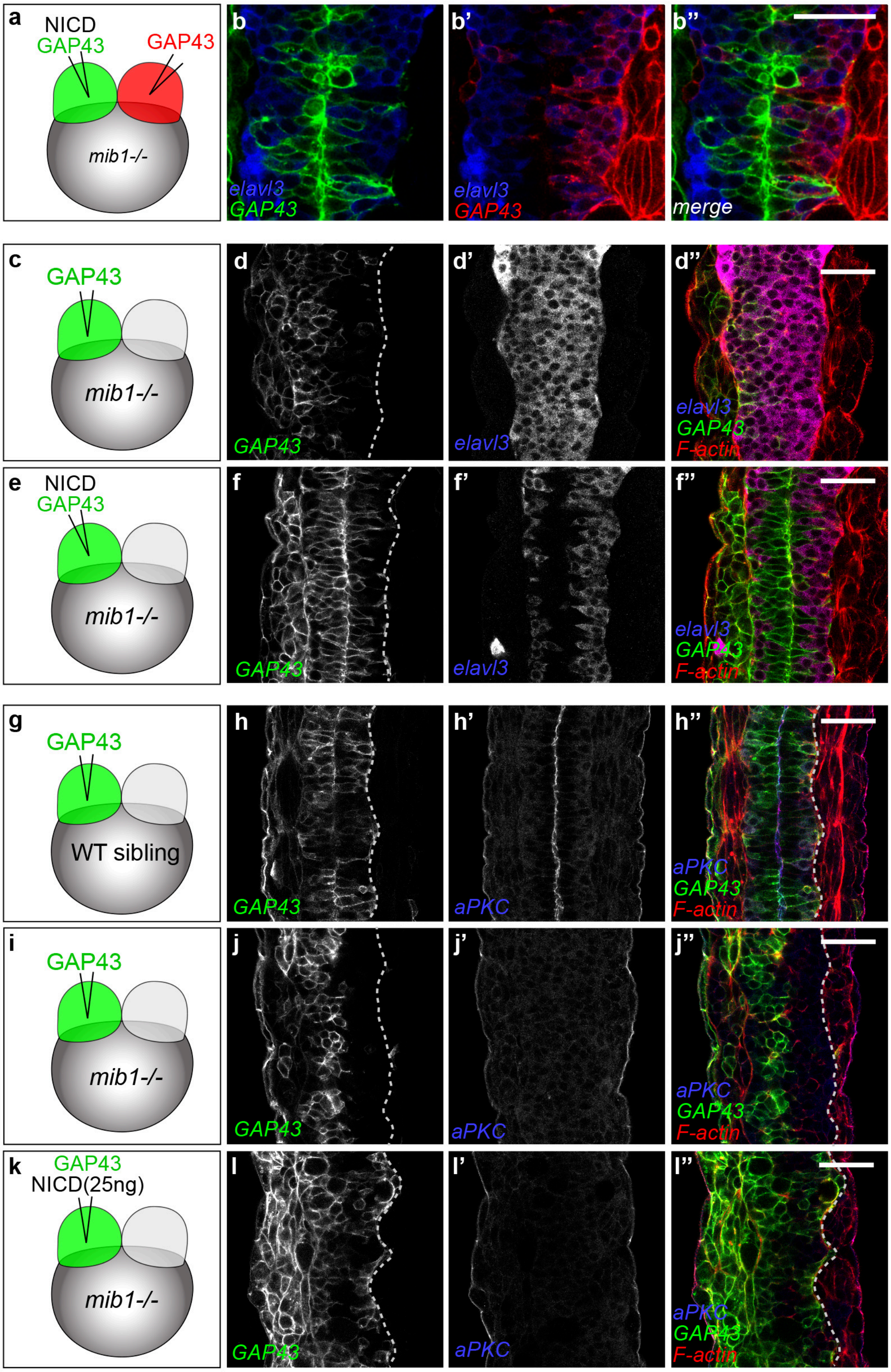

Notch-mediated suppression of neurogenesis is required for midline-crossing cell divisions in the zebrafish neural tube
(**a,b**) Activated Notch (NICD) was expressed in one half of *mib1* mutant neural tubes. NICD suppresses neurogenesis (visualized by the neuronal marker elavl3) and promotes midline-crossing in NICD-injected cells identified by green membrane label (**b**). Contralateral NICD-negative cells (red membrane label) do not cross the midline and undergo neuronal differentiation (**b'**, n=7). (**c-f**) In *mib1* mutant embryos all cells of the dorso-medial spinal cord differentiate as neurons and the exchange of cells between the two halves of the neural tube is reduced (**d,** n=10). Unilateral NICD injection results in a local suppression of neurogenesis and restores the midline crossing of elavl3-negative cells (**f**, n=10). Accordingly, the neuronal elavl3 signal occuppies a lower percentage of the neural tube in NICD-injected *mib1* mutants (f', 52.2±12.4%) than in *mib1* mutant controls (d', 83.2±9.3%, p=8.40E-06). Conversely NICD-injected *mib1* mutants present a higher midline crossing index (f, 0.86±0.05%) than *mib1* mutant controls (d, 0.63±0.05%, p=3.45E-09). (**g-l**) Unilateral injection of a low dose of NICD into *mib1* mutants fails to rescue neepithelial polarity (visualized by aPKC in **h',j',l'**) but restores the exchance of neural tube cells between the two sides of the neural tube (**h,j,l**, see Supplementary Table S3 for statistics). (**b,d,f,h,j,l**) dorsal views of the anterior spinal cord at the 18 somites (**b,h,j,l**) and 16 somites (**d,f**) stage, anterior up. Scalebars: 40 μm.

Supplementary Figure S7

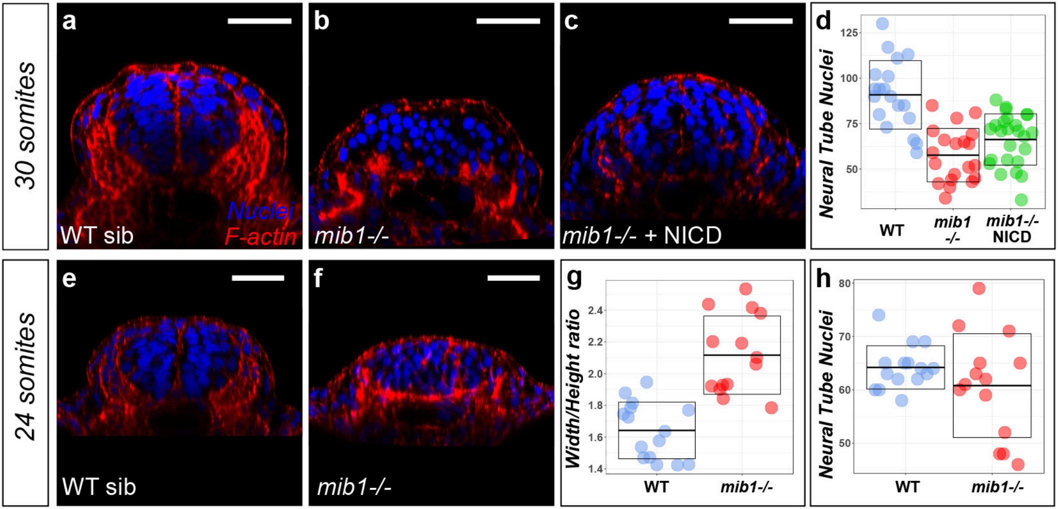

Loss of Notch signaling affects the cell number and proportions of the neural tube
(**a-d**) Nuclear Dapi staining was used to estimate the number of cells present on transversal sections of the anterior spinal cord (dorsal up, see Methods for details). The number of nuclei present in 30 somites stage *mib1* mutants (**b,d**) is reduced compared to WT siblings (**a,d**). The injection of RNA encoding constitutively activated Notch (NICD) causes a weak but not statistically significant increase in the number of *mib1* mutant neural tube cells (see Supplementary Table S5 for statistical analysis). The data set used for this analysis is the same used for the analysis of neural tube Width-to-Height ratio in **Fig. 8q**. (**a,b,c**) correspond to the display items of **Fig. 8n-p**. (**e,f,g**) At the 24 somites stage, *mib1* mutants present a significant increase in the neural tube Width-to-Height ratio (2.12±0.25, n=14) compared to WT siblings (1.64±0.18, n=15) (p=4.71E-06). (**e,f,h**) In contrast, the number of *mib1* mutant neural tube cells shows only a minor and not statistically significant decrease (60.8±9.7) when compared to WT siblings (64.2±4.1) (p=0.23). Boxes in **d,g,h** represent mean values ± SD. Scalebars: 40 µm.

Supplementary Figure S8

Supplementary Table S1:
Percentage of the neural tube area occupied by elavl3-positive neurons

	Mean value	*Standard deviation*	*Number of embryos*
mib1+/- ; mib2+/-	25.584	3.919	3
mib1-/- ; mib2+/-	74.473	11.574	10
mib1-/- ; mib2-/-	73.304	11.675	6
Test statistics for Welch's Anova			
F= 80.946	p= 1.194E-06		
p-values for pairwise comparisons (Games-Howell post-hoc test)			
	mib1-/- ; mib2+/-	*mib1-/- ; mib2-/-*	
mib1+/- ; mib2+/-	9.573E-07	1.401E-04	
mib1-/- ; mib2+/-		0.979	

Supplementary Table S2:
Midline crossing index of zebrafish neural tube cells at the 16 somites stage

	Mean value	*Standard deviation*	*Number of embryos*
Wild-type	0.952	0.024	15
mib1-/-	0.691	0.083	13
mib1-/- + NICD(37)	0.917	0.071	15
mib1-/- + NICD(25)	0.888	0.066	7
Test statistics for Welch's Anova			
F= 38.526	p= 6.622E-08		
p-values for pairwise comparisons (Games-Howell post-hoc test)			
	mib1-/-	*mib1-/-* + NICD(37)	*mib1-/-* + NICD(25)
Wild-type	1.949E-07	0.296	0.147
mib1-/-		4.031E-07	1.749E-04
mib1-/- + NICD(37)			0.792

Supplementary Table S3:
Midline crossing index of zebrafish neural tube cells at the 18 somites stage

	Mean value	*Standard deviation*	*Number of embryos*
Wild-type	0.915	0.047	8
mib1-/-	0.579	0.055	11
mib1-/- + NICD (25, half-injected)	0.818	0.083	9
Test statistics for Welch's Anova			
F= 99.889	p= 1.220E-09		
p-values for pairwise comparisons (Games-Howell post-hoc test)			
	mib1-/-	*mib1-/-* + NICD (25, half-injected)	
Wild-type	3.281E-10	0.0253	
mib1-/-		1.177E-05	

Supplementary Table S4:
Neural tube Width-to-Height ratio at the 30 somites stage

	Mean value	*Standard deviation*	*Number of embryos*
Wild-type	1.173	0.085	19
mib1-/-	2.248	0.351	20
mib1-/- + NICD(37)	1.489	0.310	25
Test statistics for Welch's Anova			
F= 93.993	p= 3.885E-14		
p-values for pairwise comparisons (Games-Howell post-hoc test)			
	mib1-/-	*mib1-/-* + NICD(37)	
Wild-type	2.565E-11	1.119E-04	
mib1-/-		1.137E-08	

Supplementary Table S5:
Neural tube cell number at the 30 somites stage

	Mean nuclei number	*Standard deviation*	*Number of embryos*
Wild-type	90.842	18.848	19
mib1-/-	57.650	14.705	20
mib1-/- + NICD(37)	66.240	14.090	25
Test statistics for Welch's Anova			
F= 18.766	p= 2.211E-06		
p-values for pairwise comparisons (Games-Howell post-hoc test)			
	mib1-/-	*mib1-/-* + NICD(37)	
Wild-type	1.819E-06	1.120E-04	
mib1-/-		0.129	
